# On the non-bonding valence band and the electronic properties of poly(triazine imide), a graphitic carbon nitride†

**DOI:** 10.1039/d3sc00667k

**Published:** 2023-04-18

**Authors:** David Burmeister, Alberto Eljarrat, Michele Guerrini, Eva Röck, Julian Plaickner, Christoph T. Koch, Natalie Banerji, Caterina Cocchi, Emil J. W. List-Kratochvil, Michael J. Bojdys

**Affiliations:** a Department of Chemistry & IRIS Adlershof, Humboldt-Universität zu Berlin Brook-Taylor-Str. 6 12489 Germany david.burmeist@googlemail.com m.j.bojdys.02@cantab.net; b Humboldt-Universität zu Berlin, Institut für Physik, IRIS Adlershof Zum Großen Windkanal 2 12489 Berlin Germany; c Institute of Physics, Carl von Ossietzky Universität Oldenburg 26129 Oldenburg Germany; d Department for Chemistry and Biochemistry, University of Bern Freiestrasse 3 3012 Bern Switzerland; e Helmholtz-Zentrum Berlin für Materialien und Energie GmbH Hahn-Meitner-Platz 1 14109 Berlin Germany

## Abstract

Graphitic carbon nitrides are covalently-bonded, layered, and crystalline semiconductors with high thermal and oxidative stability. These properties make graphitic carbon nitrides potentially useful in overcoming the limitations of 0D molecular and 1D polymer semiconductors. In this contribution, we study structural, vibrational, electronic and transport properties of nano-crystals of poly(triazine-imide) (PTI) derivatives with intercalated Li- and Br-ions and without intercalates. Intercalation-free poly(triazine-imide) (PTI-IF) is corrugated or AB stacked and partially exfoliated. We find that the lowest energy electronic transition in PTI is forbidden due to a non-bonding uppermost valence band and that its electroluminescence from the π–π* transition is quenched which severely limits their use as emission layer in electroluminescent devices. THz conductivity in nano-crystalline PTI is up to eight orders of magnitude higher than the macroscopic conductivity of PTI films. We find that the charge carrier density of PTI nano-crystals is among the highest of all known intrinsic semiconductors, however, macroscopic charge transport in films of PTI is limited by disorder at crystal–crystal interfaces. Future device applications of PTI will benefit most from single crystal devices that make use of electron transport in the lowest, π-like conduction band.

## Abstract 2

PTI nano-crystals have quenched electroluminescence. Disorder at crystal interfaces limits charge transport in PTI films. For future device applications, single crystal devices using electron transport in the lowest conduction band show promise.

## Graphical abstract

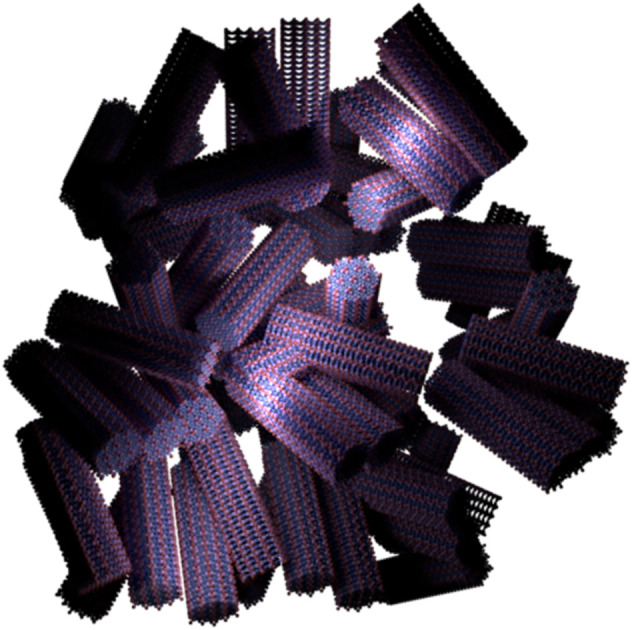

## Introduction

State-of-the-art semiconductor technology is based on rare-earth metal doped silicon that requires a lot of energy and water for its manufacture and reaches its miniaturization limits.^1–3^ In comparison, organic semiconductors are flexible, light-weight, cost-effective, and composed of earth-abundant materials. Due to their modularity, organic semiconductor materials can be tailored to have specific properties, including high carrier mobilities, low-cost fabrication, and low environmental impact. Researchers are exploring the use of organic semiconductors in a wide range of applications, from solar cells to transistors to touchscreens. However, the long-term performance of current molecular and polymeric semiconductors is low because of lacking oxidative stability and structural disorder due to the inherent degrees of freedom of their constituents.^4^ Charge carrier mobility in organic semiconductors increases as we go from 0D, aromatic molecules (≤50 cm^2^ V^−1^ s^−1^) to 1D, linear, conjugated polymers (up to 600 cm^2^ V^−1^ s^−1^).^4^ This research logically extends to covalently-bonded, layered 2D-materials often referred to as a subclass of covalent organic frameworks (COFs).^5^ In COF-type materials, strong, covalent bonds reduce in-plane disorder and weak, inter-planar van der Waals forces enable exfoliation into (atomically) thin sheets. 2D-materials, most famously graphene, have a different electronic structure than their layered analogues due to the confinement of the electron gas to the 2D-crystal.^6–8^ In general, semiconducting 2D materials are expected to suffer less from short channel effects making them uniquely suitable for highly miniaturized field effect transistors.^9^

Graphitic carbon nitrides form a subset of 2D, layered COFs that are semiconductors and can be exfoliated.^10–14^ At present, PTI and its intercalated derivatives are the best characterized graphitic carbon nitrides.^15–18^ They are composed of triazine (C_3_N_3_) units that are covalently-linked in-plane by imide-bridges into layered materials with high thermal stability up to 400 °C.^15^ Because of their high nitrogen content, PTI has a high ionization potential – that means, a high energetic difference between the vacuum level and the highest valence band – as well as a high electron affinity – meaning, a high energetic difference between the vacuum level and the lowest conduction band. As a result, PTI has a higher oxidative stability than hydrocarbon based 0D or 1D organic semiconductors.^15,19^ High quality PTI materials are typically obtained from ionothermal synthesis in eutectic melts of alkali halides resulting in intercalation of ions like Li^+^ and Br^−^ into the structure.^5,16,20–23^ Previously, we were able to reduce the amount of carbonaceous defect sites in poly(triazine imide) (Fig. S1†).^24^ In the following, we combine microscopic and macroscopic characterization methods with *ab initio* calculations to pinpoint the reasons for the surprisingly low electroluminescence and low conductivity (10^−10^ to 10^−9^ S cm^−1^) of macroscopic films of PTI.^24^

We compare two types of poly(triazine imides): PTI with intercalated LiBr (PTI-LiBr), and an intercalate-free analogue (PTI-IF). Herein, (i) we elucidate the impact of de-intercalation on the structure and electronic properties of PTI using elemental analysis, powder x-ray spectroscopy (PXRD), convergent-beam electron diffraction (CBED), electron energy loss spectroscopy (EELS), ultraviolet photoelectron spectroscopy (UPS) and solid state nuclear magnetic resonance (ssNMR), (ii) we develop a model explaining the observed red-shift of electroluminescence as well as the discrepancy between the optical gap and the band gap using diffuse reflectance spectroscopy, valence band EELS, and *ab initio* computational data, (iii) we propose an energy level diagram on the basis of density-functional theory (DFT) calculations, evaluation of the Tauc plot and UPS, and (iv) from time-domain THz spectroscopy data we find that the nano-crystalline morphology of PTI constitutes the major bottleneck for conductivity. Interestingly, our analysis based on the phenomenological Drude-Smith model reveals an unusually high charge carrier density in both PTI-materials of 10^20^ cm^−3^. The observed charge carrier concentration is independent of intercalation of ions, and it is above and beyond that of any known intrinsic inorganic semiconductor (*e.g.* Ge 2.33 × 10^13^ cm^−3^; Si 9.65 × 10^9^ cm^−3^).

To the best of our knowledge, this work establishes PTI as the first reported, organic, lone-pair semiconductor, and it will serve as a guide for even more exciting device applications of 2D, layered organic materials in the near future.

## Results and discussion

### Elemental composition

We carried out combustion elemental analysis and inductively coupled plasma-optical emission spectroscopy (ICP-OES) on both PTI derivatives to rule out carbonization and presence of residual ions in PTI-IF, and we find the results to be in good agreement with the theoretical chemical formulae (Table S1†). As observed previously in PTI-LiCl, the Li-ion loading in PTI-LiBr is too high to be explained just by intercalation of LiBr. Excess lithium isoelectronically replaces some of the hydrogen atoms at the imide bonds.^25^ Overall, we obtain a composition of [(C_3.0_N_3.0_)_2_(N_1.0_H_1−*x*_Li_*x*_)_3_·Li_1.2_Br_1.2_, *x* = 0.2] for PTI-LiBr (theoretical C_6_H_3_N_9_LiBr). Electron energy loss spectra (EELS) confirm the presence of carbon, nitrogen, lithium, and bromine in PTI-LiBr. K-edges of additional elements are not observed confirming the high purity of the product on microscopic scale (Fig. S2†).

After Soxhlet extraction and de-intercalation, we detect no Li- and no Br-ions in combustion analysis and ICP-OES. The calculated composition of PTI-IF is C_6.0_H_3.5_N_9.0_ (Li wt% ≪ 0.5) (theoretical C_6_H_3_N_9_). EELS shows no evidence of intercalated ions in the form of Li/Br K-edges at the microscopic level (Fig. S2†). In addition, we performed X-ray photoelectron spectroscopy (XPS) measurements to quantify the C : N ratio at the surface of the PTI-LiBr films. We observe a C : N ratio of 41 : 59 at% (theoretical 40 : 60 at%) (Fig. S3†). Overall, results of the macroscopic and microscopic composition analysis reflect a high material purity.

### Effect of de-intercalation on the structure and morphology of PTI crystals

**Fig. 1:**
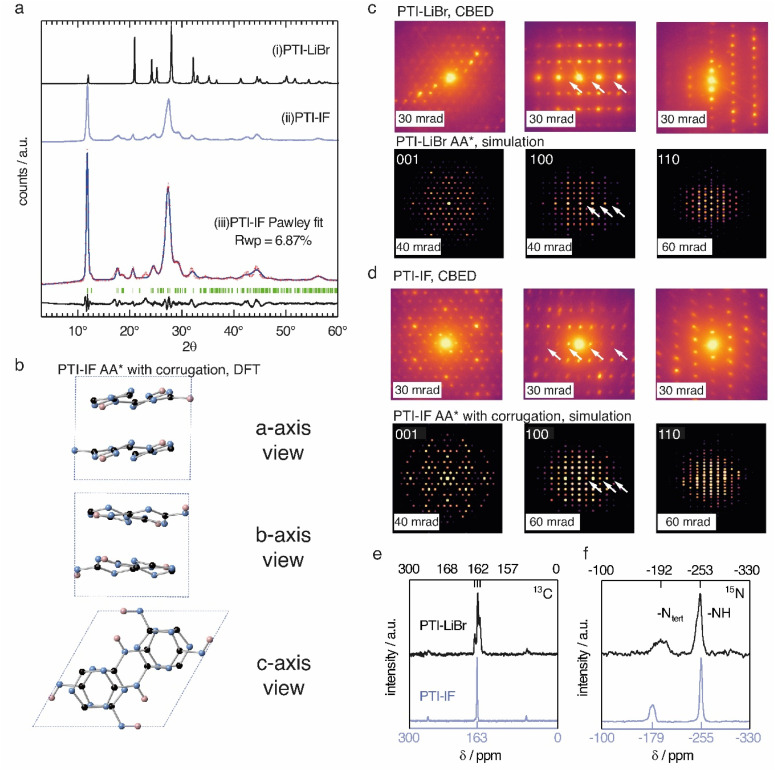
(a) Experimentally obtained PXRD patterns of (i) PTI-LiBr and (ii) PTI-IF and (iii) Pawley fit (blue line) of the experimentally obtained pattern (red crosses) to a corrugated AA* stacked PTI-IF cif file, calculated Bragg peak positions (in green), and the difference between the observed and refined pattern (in black). (b) Unit-cell of PTI-IF in a corrugated AA* arrangement based on DFT geometry optimization and Pawley fitting. The unit cell is shown along the three crystal axes with nitrogen atoms as blue spheres, carbon as black spheres, and hydrogen as pink spheres. (c) Convergent beam electron diffraction (CBED) patterns of PTI-LiBr and simulated patterns. Systematic absences marked by white arrows. (d) Experimental CBED patterns of PTI-IF and simulated patterns. (e) ^13^C and (f) ^15^N solid-state nuclear magnetic resonance (ssNMR) spectra of PTI-LiBr (black line) and PTI-IF (blue line).

The unit-cell of PTI-LiBr has been described in great detail,^18,25,26^ and it serves as point of reference for the structure refinement and solution of PTI-IF. We obtained a unit-cell for PTI-IF using powder X-ray diffraction (PXRD) (Fig. 1a), convergent beam electron diffraction (CBED) (Fig. 1d), and structural optimization by density functional theory (DFT) (Fig. 1b). The DFT-optimized unit-cell of PTI-IF agrees well with X-ray and electron diffraction data, and it indicates that intercalate-free PTI-sheets assume a corrugate arrangement.

Removal of intercalated Br-ions from PTI-LiBr – and the accompanying loss of the high-scattering cross section contribution – leads to a broadening of all X-ray diffraction peaks (Table S2†). In particular, we attribute the broadening of diffraction peaks that have 00*l*-components to the exfoliation and the decreasing height of PTI stacks. The median 002 stacking distance between PTI-sheets increases from 3.5 Å in PTI-LiBr to 3.6 Å in PTI-IF, either due to corrugation or due to intercalation of water molecules.^27^

Corrugation of triazine-based, 2D networks has been predicted computationally in the past, however, to this date there was no experimental evidence to support it.^28–30^ To corroborate that the removal of intercalated ions is indeed accompanied by corrugation and delamination, we compared the experimentally obtained diffractogram of PTI-IF with the simulated diffraction patterns of corrugated and non-corrugated PTI-structures in three different stacking arrangements: (i) alternating layers with a mirror plane through the triazine units (AA*), (ii) eclipsed orientation (AA), and (iii) staggered orientation (AB).^28–30^ Simulated diffraction patterns based on the corrugated structures match the experimental data better (*δ*Rwp = 2–7%), and they correctly describe the peak at 18° 2*θ* (assigned as −111) than the non-corrugated ones. The best Pawley fit was obtained for the corrugated PTI-IF structure in AA* stacking mode (Fig. 1a, b, S4-5 and Table S3†). Further evidence for the corrugation of PTI-IF comes from CBED (Fig. 1c and d, S6†). In the case of a perfectly planar, intercalated PTI-LiBr structure, we observe no discrepancies between observed and predicted patterns (Fig. 1c).^16^ For PTI-IF, once again the corrugated structure reproduces the experimental pattern better. In particular, simulated ED patterns based on the corrugated PTI-IF structure account correctly for the missing systematic absences in the 100 zone axis pattern (Fig. 1d and S6†). In conclusion, we arrive at a structural description of PTI-IF as a corrugated, intercalate-free, triazine-based material coming from various, orthogonal methods that encompass microscopic, macroscopic, and *ab initio* structure analysis. As the reason for the corrugation, we propose dipolar interactions of adjacent PTI-layers.^31^

Removal of the intercalated ions from PTI-LiBr has a pronounced effect on the local electronic environments of carbon- and nitrogen nuclei in PTI-IF that we monitored using solid-state NMR (ssNMR). Carbon environments attributed to the triazine (C_3_N_3_) ring are split up as ^13^C (168, 162, 157 ppm) in the PTI-LiBr structure by the heterogenous distribution of intercalated ions. In comparison, the more homogeneous, intercalate-free PTI-IF structure shows only one carbon signal at 163 ppm (Fig. 1e).^26^ Similarly, the ^15^N signals of PTI-IF appear sharper at −179 (triazine nitrogens) and −255 ppm (imide bridge nitrogen) (Fig. 1f). All ring atoms in ^15^N and ^13^C ssNMR spectra of PTI-IF are shifted to higher ppm values compared to PTI-LiBr. This finding indicates that nuclei are de-shielded by the removal of ions; an effect that stems from a reduction of electron density at the carbon and nitrogen nuclei. We corroborate this experimental observation by Bader partial charge analysis based on DFT results (Fig. S7†). In PTI-LiBr we observe an increased electron density from the interaction of bromide orbitals with the CN-orbitals.

**Fig. 2:**
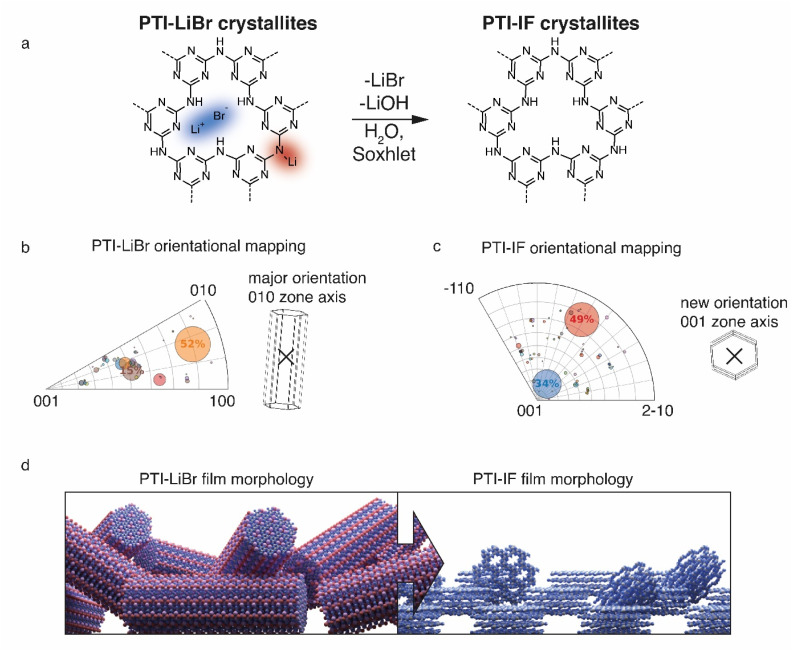
Statistical analysis of the orientations of nano-crystals of PTI-LiBr and PTI-IF based on CBED mapping. (a) Structural formula of PTI-LiBr and PTI-IF. (b) Integration of the inverse pole figure of PTI-LiBr. (c) Integration of the inverse pole figure of PTI-IF. The major observed zone axis in PTI-LiBr is 010/100. In PTI-IF it changes to 001. (d) Visualization of the different morphologies and orientations of PTI-LiBr and PTI-IF in films.

Presence or absence of intercalated ions has a pronounced effect on the preferred orientation of PTI-IF and PTI-LiBr crystals on substrates such as the carbon net used in CBED experiments. The structural formula of PTI-LiBr and PTI-IF is shown in Fig. 2a. An explanation of the orientation analysis is given in the ESI (Fig. S8).† The majority of 52% of PTI-LiBr crystals is tilted away from the 001 orientation (Fig. 2b). This means that the majority of PTI-LiBr crystals orient themselves with the long side of the pillars in parallel to the substrate and perpendicular to the incident electron beam (X) – as indicated by the wireframe model.

In contrast, for PTI-IF, a major fraction of 34% of CBED patterns is collected from crystals oriented along the 001 zone axis (Fig. 2c). This means that PTI-IF crystals are oriented with the covalently-bonded basal plane in parallel to the carbon support. The pronounced change in preferred crystal orientations is caused by the removal of ions and the accompanying reduction of cohesive, inter-planar interactions – from a mix of ionic and van der Waals interactions in PTI-LiBr to van der Waals interactions only in PTI-IF. As a result, PTI-sheets become more easily exfoliated and effectively diminish the stacking height of PTI-IF nano-crystals. The reorientation of PTI-IF nano-crystals with the covalent, delocalized planes parallel to a substrate and with pore channels orthogonal to it, is beneficial in device geometries intended for sensing and gating (Fig. 2d).

### Vibrational analysis

**Fig. 3:**
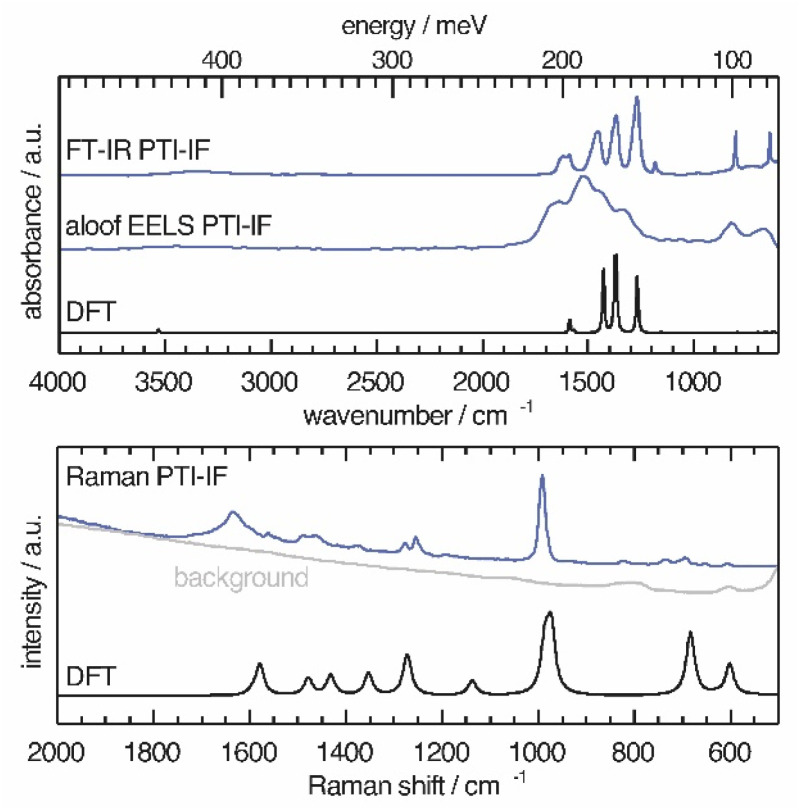
Vibrational modes in PTI-IF determined by FT-IR, aloof-EELS, DFT and Raman spectroscopy (266 nm).

We use a combination of Fourier-transform infrared spectroscopy (FTIR), Raman microscopy and electron energy loss spectroscopy (EELS) to assign phonon modes and deformation vectors to PTI-IF (Fig. 3) and PTI-LiBr (Fig. S9†).

The FT-IR spectra of PTI-LiBr have typically very broad bands (Fig. S9†). We can distinguish between the C_3_N_3_ ring mode at 808 cm^−1^ and the CN-stretches at 1100–1620 cm^−1^. Further, we observe a split of the imide N–H stretch at 3300 cm^−1^ that is caused by heterogeneously intercalated ions that break the symmetry of the material.^16,32^

The UV-Raman spectrum of PTI-LiBr shows only two distinctive features at 1629 and 982 cm^−1^. The 1629 cm^−1^ band is interpreted as C=N ring-stretches, also referred to as G band, while the band at 982 cm^−1^ represents the C_3_N_3_ breathing mode.^33^ The ring mode in PTI-LiBr has a shoulder towards higher wavenumbers. The aloof-EELS phonon spectrum of PTI-LiBr shows a broad feature reminiscent of the CN-stretches in the FT-IR spectrum. Additionally, the ring mode at around 800 cm^−1^ can be picked up with this technique.

The vibrational modes in PTI-IF are better resolved than in PTI-LiBr (Fig. 3 and S9†).^15^ DFT calculations predict the main four bands in the aloof-EELS and FT-IR spectra of PTI-IF observed between 980–1620 cm^−1^ (Fig. 3). However, the intensity of the ring modes at 642 and 802 cm^−1^ is drastically underestimated in the phonon calculation. The ring mode of PTI-IF at 990 cm^−1^ in the Raman spectrum has a higher intensity and no shoulder compared to PTI-LiBr.

The two triazine ring modes in the FT-IR spectra of PTI-LiBr are observed at 809 and 666 cm^−1^. In comparison, the ring modes of PTI-IF are observed at 802 and 642 cm^−1^. We attribute the higher energy ring-modes of PTI-LiBr to structural strain that is induced by the intercalated ions. We present the observed and calculated energies of the modes and a visual representation of the deformation vectors in an overview figure and table (Fig. S10†) to guide future interpretation of vibrational spectra of triazine-based carbon nitrides. Generally, the inhomogenous distribution of ions throughout the PTI-LiBr structure causes a significant broadening of the vibrational bands in all spectroscopic methods. Removal of the ions from the structure leads to a more homogenous chemical environment throughout PTI-IF and, hence, to narrower bands.

### Electronic structure of PTI crystals

**Fig. 4:**
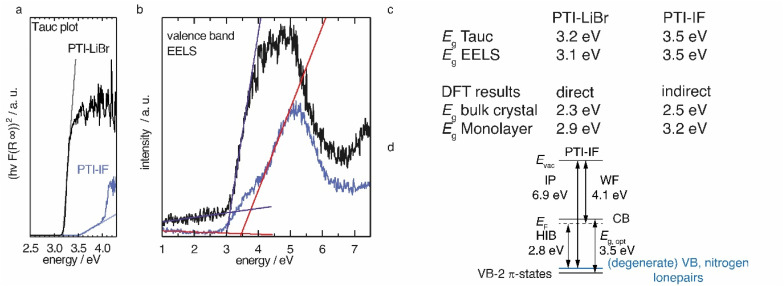
(a) Tauc plot for a direct transition of diffuse reflectance data of PTI-LiBr and PTI-IF. (b) Valence band EELS (c) summary of optical gaps extracted from Tauc plots (*E*_g_ Tauc), from valence band EELS (*E*_g_ EELS), and from DFT calculations (d) Energy level diagram of PTI-IF explaining the difference of the optical gap and the valence band-conduction band gap obtained by DFT. With the work function (WF), ionization potential (IP), the Fermi level (*E*_F_), the optical gap (*E*_g, opt_), the vacuum level (*E*_vac_) and the hole injection barrier (HIB).

We applied diffuse reflectance spectroscopy, valence band electron energy loss spectroscopy (VB-EELS) (Fig. 4), DFT (Fig. S11†), and UPS (Fig. S12†) to determine the size (eV), the nature (direct or indirect), and the energetic position of the band gap of PTI-type materials. We determined the optical gap of nano-crystals of PTI-materials using the onset values from reflectance spectra (Tauc plot, Fig. 4a),^34,35^ and valence band-EELS spectra of the conduction band (Fig. 4b). The DFT calculations show that the valence band of a monolayer is doubly degenerate at the *Γ*-point, and that it is composed of states that contain contributions from the nitrogen lone-pairs (Fig. S11†). Group-VI semiconductors with a lone-pair upper most valence band are referred to as “lone-pair semiconductors”.^36^ The non-bonding character of the VB has been theoretically predicted for other graphitic carbon nitride materials.^31^ In CN-containing molecules like melamine, both the highest occupied orbital (HOMO) and the lowest occupied orbital (LUMO) have π-character; thus, the HOMO–LUMO transition is allowed.^37^ However, in PTI layers the electronic VB → CB transition is forbidden because there is no orbital overlap between the non-bonding orbitals of the VB and the π*-like orbitals of the conduction band (Fig. S11a†). We observe a similar behavior in the bulk assembly of PTI layers, corrugation only lifts the degeneracy at the *Γ*-point (Fig. S14b†). The forbidden VB → CB transition prevents direct estimation of the band gap by optical methods or EELS as the experimentally observed absorption corresponds to the π → π* transition between the VB-2 and the CB. The energetic difference between the forbidden VB → CB and the observed VB-2 → CB transitions is the reason for the observed discrepancy between the DFT band gap and the experimental absorption onset obtained from the Tauc plot and EELS measurements (Fig. 4a and b). The VB-2 → CB transition is a π → π* transition of 3.6 eV that corresponds more closely with the experimentally derived gap by Tauc plot and aloof-EELS (Fig. 4c). We summarize the results of DFT, EELS, and UPS considerations in an energy level diagram (Fig. 4d).

We observe that the π-band (VB-2) lies below the VB and, as a consequence, the energy of the CB is lowered, *i.e.* it lies closer to the Fermi level. The energetic distance between VB and VB-2 in the intercalation free monolayer of PTI-IF is approx. 0.3 eV at *Γ* (Fig. S11a†) and the CB position (or electron affinity) is 3.7 eV. Repeating this calculation for PTI-LiBr, we obtain an electron affinity of 4.2 eV; a value that allows environmentally stable n-transport.^38^

To estimate the strength of the VB-2 → CB transition, we determined the absorption coefficients of PTI-IF and PTI-LiBr. The obtained absorption coefficients point towards a direct π → π* (VB-2 → CB)-transition in both materials (9032 M^−1^ cm^−1^ for PTI-IF, and 6860 M^−1^ cm^−1^ for PTI-LiBr, Fig. S13†), and they are one to two magnitudes lower than the absorption coefficients of molecular species without a forbidden HOMO–LUMO transition.^39,40^

Comparing PTI-LiBr and PTI-IF, removal of intercalated ions induces a significant blue shift of the absorption onset by 0.3 eV (from 3.2 eV in PTI-LiBr to 3.5 eV in PTI-IF), as seen in the Tauc plot (Fig. 4a). This increase of the optical gap is also present – but less apparent – in the EELS valence band spectra (Fig. 4b). The shoulder appearing below the main EELS band is likely a result of beam damage as it grows in intensity during the measurement. The gap increases from 3.1 eV (PTI-LiBr) to 3.5 eV (PTI-IF) by VB-EELS. The lower optical gap and band gap of PTI-LiBr can be ascribed to two concomitant mechanisms: (1) the formation of ion pairs (*i.e.* static dipoles) within the pores from the intercalated species that break the symmetry of the system. Eventually, this leads to the splitting of the degenerate levels at *Γ*; (2) the presence of intra-optical-gap defect states associated to the p orbitals of Br anion (Fig. S13a†).

PTI-type intercalation compounds reported hitherto in literature typically show lower optical gaps (2.2–2.7 eV) due to carbonization.^17^ The presence of a non-bonding band above the π-band (Fig. 4d) is significant as it does not allow for effective carrier injection into the π-band. This leads to the absence of the photoluminescence band in the electroluminescence spectrum and, hence, to the defect dominated electroluminescence spectrum as well as the low luminance of the organic light emitting device (OLED).^24^ Holes injected into the VB will be trapped due to the nature of the highly localized lone-pair orbitals and recombination will be forbidden. To complement the understanding of the electronic structure with data characterizing the electronic carriers we performed time domain-THz spectroscopy.

### Charge transport properties of nano-crystalline PTI

Intrinsic (*i.e.* undoped) organic semiconductors typically have a charge carrier density of 1 cm^−3^ and low mobility, as charge carriers are localized at single molecular units leading to hopping as dominant transport mechanism.^41^

Nano-crystalline PTI-IF and PTI-LiBr films from nano-crystals typically show low conductivity (PTI-IF 10^−10^ S cm^−1^, PTI-LiBr 10^−7^ S cm^−1^) and only small photo-currents in lateral thin-film devices.^24^ To answer the question whether the low conductivity of PTI-films is an intrinsic material property or whether it is induced by the nano-crystalline character of the sample, we subjected free standing pressed pellets of PTI-LiBr and of PTI-IF to time domain-terahertz spectroscopy (THz-TDS) experiments.

**Fig. 5:**
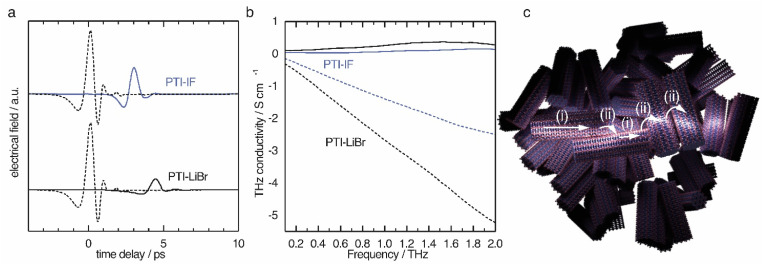
THz-time domain spectroscopy results of PTI-IF and PTI-LiBr as pressed pellets. (a) THz reference pulse (dashed lines) transmitted through PTI-IF (blue) and transmitted through PTI-LiBr (black). (b) Real (continuous line) and imaginary part (dashed line) of THz conductivity in PTI-IF (blue) and PTI-LiBr (black). (c) Schematics of charge transport in PTI thin films. Nanoscale conductivity inside nano-crystals as obtained from THz spectroscopy is up to eight orders of magnitude higher than the electrical conductivity obtained from thin film measurements. The reason is that the charge transport between the nano-crystals (ii) is inefficient compared to the charge transport inside the crystals (i) probed by THz spectroscopy.

The obtained time domain THz pulses after transmission through the sample (Fig. 5a) solid lines show a significant difference in amplitude and a phase shift when compared to the pulse that did not pass the sample (dashed lines). The observed shifts contain information about the charge carriers inside the nano-crystals. Since the THz pulse is of picosecond duration, it can be translated to the conductivity on the nanoscale by a fast Fourier transform as explained in detail in the ESI.†

The THz conductivity at 1 THz is 0.26 S cm^−1^ in PTI-LiBr and 0.07 S cm^−1^ in PTI-IF, exceeding the macroscopic electrical measurements by about six and eight orders of magnitude, respectively (Fig. 5b). Hence, the conductivity inside the single crystals of PTI-LiBr and PTI-IF is much higher (Fig. 5c(i)) than in the films. This is most likely due to inefficient charge transport between loosely packed PTI crystals (Fig. 5c(ii)) in the macroscopic films.^42^ We surmise that the reason for the higher THz conductivity in PTI-LiBr compared to PTI-IF lies in the higher orbital coefficients on the lithiated imide bridges and, as a consequence, better charge transport between the triazine units (Fig. S11c†).

We analyzed the obtained complex THz conductivity using the Drude-Smith model (DSM) that describes Drude-type transport of free charges impeded by spatial confinement with a second term including a localization parameter (Fig. 5b and S15b†).^43^ The phenomenological DSM gives an estimation of the density of charge carriers *N*, the scattering time *τ*, and the localization parameter *c*, which lies between 0 and −1 that describes Drude-type transport and completely immobile charge carriers, respectively.^44^ The fitting parameters reveal a charge scattering time of 2.5 and 2.9 femtoseconds for PTI-LiBr and PTI-IF, respectively, and a localization parameter of <−0.999 for both (Fig. S15c†). The low scattering time as well as the localization parameter close to −1 imply a high degree of localization. Using the hole effective mass obtained from the DFT calculations (PTI-LiBr *m*_eff_ = 2.25, PTI-IF *m*_eff_ = 1.20), the carrier mobility in the nanoscale *μ*_THz_ and the effective mobility *μ*_eff_ can be calculated as described in the SI. The effective mobility of 10^−3^–10^−4^ cm^2^ V^−1^ s^−1^ of PTI-LiBr and PTI-IF is in the range of the one found in amorphous organic semiconductors.^45^ The reasons for the low mobility is the absent conjugation along the imide bridge as well as the highly localized character of the valence states.

The density of charge carriers contributing to the nanoscale conductivity in PTI-LiBr (3.00 × 10^20^ cm^−3^) is orders of magnitude higher than what would be expected from an organic semiconductor with a large band gap and no doping.^46^ The ion free PTI-IF has a similar charge carrier density (2.07 × 10^20^ cm^−3^) revealing that the ions are not responsible for generating electronic carriers in the organic crystals.

The obtained charge carrier density is higher than observed for other layered crystals such as covalent organic frameworks, metal organic frameworks and transition metal di-chalcogenides, for example MoS_2_ (Table S4†). The carrier densities closest to PTI are those of graphite and MoS_2_ bulk crystals (1 × 10^19^ cm^−3^ and 3.7 × 10^19^ cm^−3^, respectively).^47–49^ The high carrier concentration might either be generated by an unknown structural defect or by adsorbed molecules like water or oxygen. Symmetric PTI devices with interdigitated indium-tin oxide electrodes are indeed sensitive to pressure, pointing towards water or oxygen acting as adsorbed dopants under environmental conditions (Fig. S16†).^27^

## Conclusion

We present an in-depth analysis of the structure, the orientation, and the electronic properties of a graphitic carbon nitride – PTI, with and without intercalated lithium bromide – and we provide a comprehensive energy level diagram for this fascinating material class.

We find that the band gap of the material cannot be assessed directly by optical methods because the VB → CB transition is forbidden. The non-binding upper-most valence band is likely the reason for the previously reported low electroluminescence. As a consequence, we find that PTI is not viable as emission layer in efficient organic light emitting diodes.

Using time-domain THz spectroscopy, we find that – despite its covalent character and high crystallinity – PTI experiences a high charge carrier localization and low mobility. The low conductivity in macroscopic films is caused by the nano-crystalline morphology. Removal of ions leads to a decreased electron density at the carbon and nitrogen cores as well as layer corrugation.

Future device architectures should exploit the highly stable and porous character of PTI that could be used for ion migration and retention at semiconducting interfaces. Such applications can make use of the preferred orientation effects we observed in this study, as pore-channel entrances of PTI-IF nano-crystals align perpendicular to a flat host-substrate. Further, once PTI single crystals of about 50 μm or larger are available, the extraordinarily stable n-type transport characteristics could be studied in single-crystal devices.

## Data availability

Additional information availabe at: https://doi.org/10.26434/chemrxiv-2023-dt7vk-v2.

## Author contributions

D. B. designed the experiments, conducted them, collected the experimental data and interpreted it. Theoretical analysis and interpretation was conducted by M. G. and C. C.. E. R. and N. B. conducted the THz spectroscpy study, interpreted the results and wrote the corresponding text. J. P. contributed UV-Raman results. A. E. and Ch. T. K. designed the electron microscopy diffraction studies, collected and interpreted the data and wrote the corresponding text. D. B. drafted the article with inputs from all authors. M. J. B. and E. L. K. revised the manuscript.

## Conflicts of interest

There are no conflicts to declare.

## Supplementary Material

SC-014-D3SC00667K-s001
