# Supplementary material for: On the non-bonding valence band and the electronic properties of poly(triazine imide), a graphitic carbon nitride
Source: Chem Sci. 2023 Apr 18;14(23):6269–77. doi: 10.1039/d3sc00667k (PMC10266476; doi:10.1039/d3sc00667k)
Supplement: SC-014-D3SC00667K-s001 [file SC-014-D3SC00667K-s001.pdf]

Electronic Supplementary Material (ESI) for Chemical Science.
This journal is © The Royal Society of Chemistry 2023

Supporting Information

On the non-bonding valence band and the electronic properties of poly(triazine imide), a graphitic carbon nitride

David Burmeister, Alberto Eljarrat, Michele Guerrini, Eva Röck, Julian Plaickner, Christoph T. Koch, Natalie Banerji, Caterina Cocchi, Emil J. W. List-Kratochvil, Michael J. Bojdys**

Experimental

Experiments **have to be conducted with caution** since they employ glassware under high pressure and temperature.

Preparation of PTI-LiBr: 1 g of precursor dicyandiamide (DCDA, Sigma Aldrich >99%) is ground together with an eutectic salt mixture of LiBr and KBr (Sigma Aldrich /Acros Organics >99%) (15 g; 58:42 wt%, m.p. 348 °C) in a glovebox. The reaction mixture is filled into a quartz ampule and sealed. The sealed quartz ampule is placed into a furnace (Nabertherm, L 5/11/B180, 2.4 kW) at 400 °C for 4 h. Then the temperature is increased (10 K/min) to 475 °C for 7 days. The furnace is then allowed to cool naturally. The ampule is removed at room temperature, opened, and the salt block is dissolved in Milli-Q water in a 50 mL Falcon. The slurry is centrifuged and the supernatant is decanted. The pellet is dispersed in hot water on a shaker and centrifuged again. The supernatant is decanted and the process is repeated for two times with hot water and two times with methanol (>99% for synthesis). The resulting pellet is re-dispersed in methanol, transferred into vacuum safe glas ware and the methanol is evaporated. The resulting powder is dried under vacuum at 200 °C for 24 h before combustion analysis. The yield is 82% (0.93 g).

Preparation of PTI-IF: 0.7206 g PTI-LiBr is filled into a Soxhlet thimble and extracted for 20 days with water. To retain crystallinity the product is only dried at 60 °C at ambient pressure

for 24 h. Drying at 200 °C in reduced pressure reduces crystallinity. The yield is 0.4699 g, 94%.

Preparation of suspensions for TEM, SEM, Raman sample preparation: For the dispersion 10-20 mg of product are re-dispersed in 5 mL of EtOH. The dispersion is then dropcast either on a TEM grid (Plano, S147-2, holey carbon) or a wafer (1 µL) or a 1x1 cm quarz glas substrate for Raman (10-50 µL).

PTI suspensions: 6 mg PTI are suspended in 1 mL methanol and pipetted up and down.

Fourier transform infrared (FT-IR): Spectra were recorded from solid on a Thermo Scientific Nicolet iS5 spectrometer (Thermo Fisher Scientific, Waltham, MA, USA) in the wavenumber range of 4000-600 cm^{-1} with resolution of 4 cm^{-1}.

DFT calculations: The PTI-IF (bulk and monolayer) structure was taken from Ref. [DOI: 10.1039/c2ta01068b] removing the Li and Br ions and adding hydrogen to the imide bridges. The structure was subsequently optimized using the PBE exchange-correlation functional with the Tkatchenko-Sheffler pairwise correction scheme for van der Waals dispersions, a plane wave cut-off of 50 Ry, norm-conserving pseudo-potentials, and a 6x6(x6) Monkhorst-Pack k-mesh to ensure convergence of the electronic band structure.[1–3] The PTI-LiBr (bulk and monolayer) has been obtained from the structural relaxation of the geometry taken from Ref.[DOI: 10.1039/c2ta01068b] using the same computational parameters as specified above. Both the PTI-IF and PTI-LiBr 2D structures (monolayers), on which phonon calculations have been performed, have been obtained from the bulk structures by taking out one single layer (and associated LiBr intercalants) and adding 20 Å of vacuum in the normal direction with respect to the monolayer plane in order to avoid spurious interaction between the replicas upon the application of periodic boundary conditions. All calculations were performed with the ab initio package- Quantum Espresso.[4]

To study the effect of lithium replacing hydrogen at the imide bridges, one third of the

hydrogens in the PTI-IF monolayer was replaced by lithium and the structure was further

Unit cell data of DFT optimized AA* stacked PTI-IF.

```
# CRYSTAL DATA

#-----------------------------------------------------------------------

data_VESTA_phase_1


_chemical_name_common                  'XCrySDen XSF file'
_cell_length_a                         8.02801
_cell_length_b                         8.16505
_cell_length_c                         6.34309
_cell_angle_alpha                      90.70694
_cell_angle_beta                       89.98965
_cell_angle_gamma                      119.43531
_space_group_name_H-M_alt              'P 1'
_space_group_IT_number                 1

loop_
_space_group_symop_operation_xyz
   'x, y, z'

loop_
   _atom_site_label
   _atom_site_occupancy
   _atom_site_fract_x
   _atom_site_fract_y
   _atom_site_fract_z
   _atom_site_adp_type
   _atom_site_B_iso_or_equiv
   _atom_site_type_symbol
   C1       1.0     0.683936      0.167584      0.301117     Biso  1.000000 C
   N1       1.0     0.525734      0.176754      0.345431     Biso  1.000000 N
   N2       1.0     0.676592     -0.001833      0.285344     Biso  1.000000 N
   C2       1.0     0.856113      0.473902      0.249480     Biso  1.000000 C
   C3       1.0     0.542938      0.339775      0.283267     Biso  1.000000 C
   C4       1.0     0.315150      0.837415      0.767967     Biso  1.000000 C
   C5       1.0     0.142820      0.530788      0.817544     Biso  1.000000 C
   C6       1.0     0.456007      0.665016      0.784318     Biso  1.000000 C
   C7       1.0     0.797478      0.348459      0.783968     Biso  1.000000 C
   C8       1.0     0.484186      0.176262      0.800347     Biso  1.000000 C
   C9       1.0     0.618433      0.482406      0.748672     Biso  1.000000 C
   C10      1.0     0.201349      0.656444      0.284051     Biso  1.000000 C
   C11      1.0     0.514689      0.828717      0.268841     Biso  1.000000 C
   C12      1.0     0.380228      0.522179      0.318190     Biso  1.000000 C
   N3       1.0     0.856118      0.313057      0.273851     Biso  1.000000 N
   N4       1.0     0.699926      0.487003      0.208312     Biso  1.000000 N
   N5       1.0     0.473614      0.828629      0.723998     Biso  1.000000 N
   N6       1.0     0.142778      0.691626      0.793109     Biso  1.000000 N
   N7       1.0     0.298910      0.517521      0.858772     Biso  1.000000 N
   N8       1.0     0.787934      0.495709      0.708747     Biso  1.000000 N
   N9       1.0     0.651355      0.185625      0.846087     Biso  1.000000 N
   N10      1.0     0.457333      0.321456      0.771033     Biso  1.000000 N
   N11      1.0     0.210778      0.508883      0.358521     Biso  1.000000 N
   N12      1.0     0.347607      0.819841      0.223661     Biso  1.000000 N
   N13      1.0     0.541403      0.683169      0.296046     Biso  1.000000 N
   N14      1.0     0.024512      0.639716      0.256722     Biso  1.000000 N
   N15      1.0     0.382394      0.356168      0.308374     Biso  1.000000 N
   N16      1.0     0.322304      0.006640      0.785932     Biso  1.000000 N
   N17      1.0    -0.025646      0.365030      0.810382     Biso  1.000000 N
   N18      1.0     0.616222      0.648351      0.757874     Biso  1.000000 N
   H1       1.0     0.020485      0.754709      0.205959     Biso  1.000000 H
   H2       1.0     0.802548     -0.001731      0.285475     Biso  1.000000 H
   H3       1.0     0.196334      0.006487      0.784564     Biso  1.000000 H
   H4       1.0    -0.021608      0.250153      0.861591     Biso  1.000000 H
   H5       1.0     0.263399      0.241162      0.359132     Biso  1.000000 H
   H6       1.0     0.735521      0.763467      0.707939     Biso  1.000000 H
```

optimized.

IR absorption spectra and phonon normal modes for the PTI-IF and PTI-LiBr monolayers have been calculated in the framework of density functional perturbation theory with the *PHonon* package of Quantum Espresso using a 6x6 q-mesh.

Calculated Raman spectra were obtained using CASTEP as embedded in the Materials Studio 7.0 suite after a geometry optimization and vibrational analysis using the PBE as exchange-correlation functional, a plane-wave basis set cut-off of 700 eV, and choosing an excitation source with wavelength 266 nm.[5]

Raman: UV Raman spectra were recorded with a Horiba T64000 spectrometer in single-grating mode. The excitation source was provided by a diode-pumped solid-state laser from CryLas at a wavelength of 266 nm and power of 4 mW. The light was focused on the sample with a Thorlabs LMU-40x-UVB objective (backscattering geometry). A notch filter with cut-off at 220 cm^{-1} was used to filter out the elastically scattered light. The acquired Raman spectra were calibrated by comparison with the spectrum of a Ga_2O_3 crystal by using a quadratic calibration curve. This procedure allows for reduction of the experimental error at approximately 5 cm^{-1}, which is below the spectral resolution of 8 cm^{-1}.

Powder X-ray diffraction (PXRD): Structural analysis of the prepared PTI-MX was performed with a Bruker D2 Phaser X-Ray powder diffractometer (XRD) in Bragg-Brentano geometry. X-rays were generated by a Cu $K_{\alpha1+2}$ source at 30 kV operating voltage and collected with a LynxEye detector.

Optical microscopy: Film thickness measurements for determination of conductivity were performed with an Olympus LEXT laser scanning microscope.

ssNMR: Cross polarization magic-angle spinning (CP-MAS) solid-state NMR spectra were recorded on a Bruker Avance 400 MHz spectrometer operating at 100.6 MHz (^{13}C) and 40.52 MHz (^{15}N).

EELS, CBED: Scanning transmission electron microscopy (STEM) studies were performed for the intercalation-free PTI-IF and LiBr intercalated PTI-LiBr samples. Scanning electron energy-loss spectroscopy (EELS) and convergent beam electron diffraction (CBED) experiments were carried out. All data was acquired using a Nion HERMES microscope equipped with an aberration corrector, a cold-field-emission-gun (cFEG), a monochromator at ground potential, and a hybrid-pixel direct-detection camera (Dectris ELA). The accelerating voltage was 60 kV and other experimental conditions were adjusted to each case as detailed below.

For EELS experiments, medium STEM convergence and collection angles (20 mrad) were matched in order to have good spatial resolution (130 pm) and discard the scattered beams from entering the EELS detector. This probe is rastered over the sample collecting an EELS spectrum for each position. Aloof and transmission EELS was carried out to assess induced damage to the beam sensitive sample.

In CBED mode, a relatively small convergence angle (0.5 mrad) of the STEM probe was used in order to form smaller, point-like diffraction disks. This probe is rastered over the sample collecting a CBED pattern for each position. In order to obtain CBED signal, the beam needs to transmit through the sample, cancelling the possibility of an aloof acquisition mode.

The acquired CBED datasets are large (several Gb), since the diffraction pattern dimensions are 512x512 pixel and the raster dimensions are 100x100 pixel. In order to analyze them, the open-source Python library `pyxem` in combination with `hyperspy` were employed.[6–8]

Distortion of the experimental diffraction data was corrected by measuring the elliptical shapes of rings in position averaged CBED and house-developed software.

Pawley refinement: Pawley refinement of the unit cell was performed using the Reflex tools in the Materials Studio 7.0 suit.

UV-Vis of dispersions: UV-VIS measurements were performed in ambient conditions with a PerkinElmer Lambda 950 spectrometer in standard transmission operation with 1 nm step size. Dispersions were obtained by dispersing a known quantity of PTI powder in DI water and sonicating it for 60 min with a benchtop sonication bath. Dispersions were measured in a quartz cuvette with 1 cm optical path. Molar absorption coefficients were obtained equating the mass of one unit cell with one molecule, using Lambert-Beer law.

$$A = \varepsilon\, c\, d \#(1)$$

A: absorbance

c: concentration

d: length of the optical path in cuvette

ε: molar absorption coefficient

Solid state UV-Vis: A Edinburgh Instruments FLS 980 spectrometer was used. Reflectance spectra were collected with a Xe lamp in an Ulbricht sphere. The reflectance spectra were converted using the Kubelka-Munk relation and plotted via the Tauc expression.

Kubelka Munk equation:

$$F(R\infty) = \frac{(1 - R(\infty))^2}{2\, R(\infty)} \#(2)$$

F(R∞): Kubelka Munk function

R(∞): reflectance of infinite thick material

Relation proposed by Tauc Mott and Davis for direct band gaps:

$$C\left(hv - E_g\right) = (F(R\infty)\ hv)^2 \#(3)$$

C: proportionality constant

h: plank constant

v: frequency

E_g: band gap energy

Preparation of thin film devices for conductivity determination: Ossila “Interdigitated ITO Substrates for OFET and Sensing” S161: w × L: 30 mm × 50 µm were coated with a PTI-LiBr and PTI-IF dispersion by drop-casting. Samples were placed in an Ossila low density OFET test board and IV characterisation was conducted with a source measure unit (4200A SCS parameter analyser; Keithley).

$$\sigma = \frac{1}{\rho} = \frac{L}{\frac{U}{I}\,A} = \frac{L}{\frac{U}{I}\,w\,t} \#(4)$$

⍰= electrical resistivity
σ=electrical conductivity
U=bias
I=current
A=surface area
w=channel width
t=film thickness
L=channel length

Devices for pressure sensitivity measurements were placed in a vacuum chamber and the pressure was reduced with a laboratory vacuum oil pump.

THz-spectroscopy: The THz time domain spectroscopy (THz-TDS) setup is based on the output of a regeneratively-amplified Ti:sapphire laser system (Coherent Astrella). Part of the 800 nm output with an average power of 6.5 W and ~35 fs pulse duration at 1 kHz repetition rate is split to a 30 μW beam to generate the probe beam. The THz probe pulses are

generated via optical rectification in a 2 mm [110] ZnTe crystal. The THz probe pulse is detected by electro-optic sampling in another 1 mm [110] ZnTe crystal. The setup from the ZnTe generation crystal, sample holder, to the detector camera is enclosed in a chamber in nitrogen atmosphere to exclude THz absorption by air humidity. The immediate response of the THz electric field to the charges in the investigated material yields the ground state conductivity. It is obtained from the Fourier transform of the THz probe response to the pressed pellets and the Fourier transform of the transmitted THz pulses through air.

To get information about the charge carrier mobility, the conductivity spectra are fit using the modified Drude–Smith Model (DSM). The experimental real and imaginary conductivity σ as a function of THz frequency ω are fitted well using the DSM and give a valuable estimation of the number of charge carriers per unit volume N, the average undisturbed travelling time τ and the localization parameter c.

$$\sigma(\omega) = \frac{Ne^2\tau/m^*}{1-i\omega\tau}\left(1+\frac{c}{1-i\omega\tau}\right) \#(5)$$

Hereby, the effective mass m^* of the holes is used from density functional theory calculations of PTI-LiBr and PTI-IF. The localization parameter c can vary between 0 and -1, where the charge transport is Drude-like and completely suppressed, respectively. The localization parameter c stems from factors such as structural disorder, coupling to nuclear modes and interactions with ions and/or between the charge carriers. It is important to highlight that the localization parameter describes the overall localization of charges but does not distinguish between the aforementioned factors.

We use τ and c to calculate the effective THz mobility μ_{eff}, which includes the localization parameter, for the pressed pellets of PTI-LiBr and PTI-IF. It corresponds to the mobility over distances probed by THz measurements, i.e. few nanometers.

$$\mu_{eff} = \mu_{THz}(1 + c) = \frac{e\tau}{m^*}(1 + c) \quad ,\#(6)$$

where the effective mass m^* of the holes is determined by DFT calculations. The THz mobility μ_{THz} describes mainly the coherent charge motion in a nano-crystal. We find the effective mobility in PTI-LiBr ($1.81 \cdot 10^{-3}$ $cm^2V^{-1}s^{-1}$) to be significantly higher than in PTI-IF ($7.33 \cdot 10^{-4}$ $cm^2V^{-1}s^{-1}$).

XPS, UPS: The samples were prepared by drop-casting PTI dispersions (MeOH) on ITO coated glass substrates to minimize charging and to avoid background carbon signals from e.g. carbon tape as substrate. For UPS experiments, a hydrogen discharge lamp (employing H Lyman-α lamp from Excitech, 10.2 eV) in a JOEL JPS-9030 Photoelectron Spectrometer was used.(doi: 10.1016/j.orgel.2016.11.032) For determination of the secondary electron cut off (SECO) a negative bias of 10 V was applied to clear the analyzer work function. All measurements were performed at room temperature. The obtained values were rounded to one decimal place. The work function was determined by linear fitting of the background and the linear region of the SECO, and reading of the point of intersection. The hole injection barrier was determined by linear fitting of the background and the valence band onset, and reading of the point of intersection. The error of the measurement is ±0.1 eV.

For XPS the JOEL JPS-9030 Photoelectron Spectrometer with an Al kα (1486 eV) excitation source and a monochromator was utilized. Quantitative comparison of nitrogen and carbon in single spectra were performed by signal integration after background subtraction of a Shirley function.

Table S1. Theoretical and experimentally determined elemental analysis of PTI-LiBr and PTI-IF in wt% from combustion analysis and ICP-OES.

	C	N	H	Li	residual wt%
PTI-LiBr	24.67	43.02	0.87	4.47	26.95
$C_6H_3N_9LiBr$ PTI-LiBr in theory	25.02	43.77	1	2.41	27.75
PTI-IF	35.00	61.53	1.73	0	1.74
$C_6H_3N_9$ PTI-IF in theory	35.83	62.67	1.51	0	0

Table S2. Peak positions and FWHM of hk0 and 00l peaks of PTI-IF and PTI-LiBr.

hkl.	PTI-IF peak position, FWHM	PTI-LiBr peak position, FWHM
010	11.82 2θ, 0.37 2θ	11.99 2θ, 0.11 2θ
110	20.56 2θ, 0.68 2θ	20.88 2θ, 0.12 2θ
002	24.71 2θ, 0.93 2θ	25.14 2θ, 0.23 2θ

Table S3. Pawley refinement of cif files with different stacking orders and with and without layer corrugation.

	Flat Rwp%	Corrugated Rwp%
AA*	17	7
AA	20	13
AB	14	12

Table S4. Comparison of the THz analysis of PTI to other layered materials in literature.

Layered material	charge carrier density (298 K)	THz conductivity (S cm^{-1})	mobility (298 K, varying methods) ($cm^2 V^{-1}s^{-1}$)
graphite[9,10]	$1\cdot10^{19}$ cm^{-3}		10^{-4}
MoS_2 bulk crystal[11]	$3.7\cdot10^{19}$ cm^{-3}		
CuPc-MIDA-COF[12]	$2.6\cdot10^{12}$ cm^{-3}	0.01	13.3 ± 7.5
ZnPc-pz and CuPc-pz COF[13]	10^{12} cm^{-3}	$5\cdot10^{-7}$	5
TPB-TFB COF[14]	3−5 · 10^{16} cm^{-3}		165 ± 10
$Fe_3(THT)_2(NH_4)_3$ MOF [15]	10^{16} cm^{-3}	0.8	220
Doped polymer poly(3-hexylthiophene)[16]	10^{21} cm^{-3}		
PTI-LiBr (this work)	$3\cdot10^{20}$ cm^{-3}	0.26	$1.81\cdot10^{-3}$
PTI-IF (this work)	$2\cdot10^{20}$ cm^{-3}	0.07	$7.33\cdot10^{-4}$

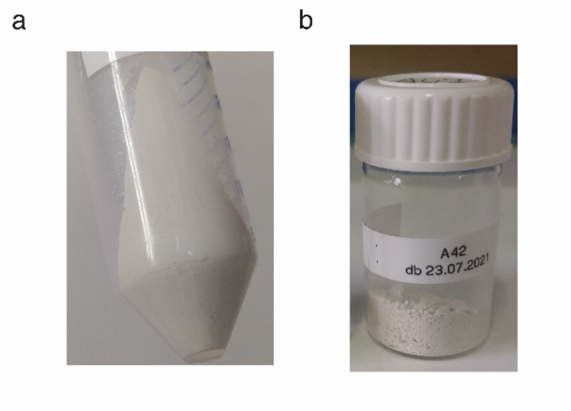

Figure S 1. Images of PTI-LiBr synthesized at 475 °C, 7 d. a) pellet after centrifugation in water. b) powder after drying.

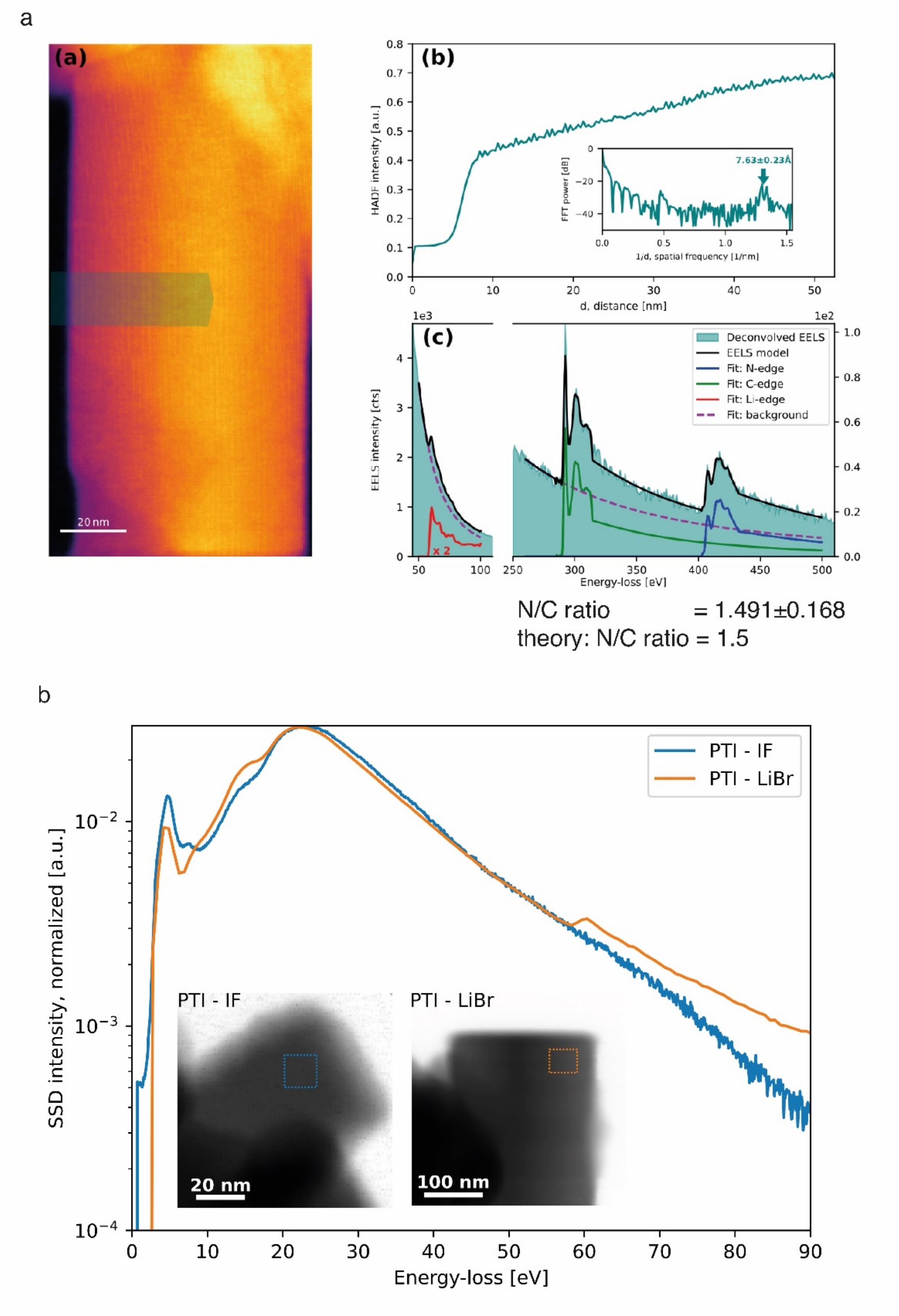

Figure S 2. a) (a) High angle annular dark-field (HAADF) image of the PTI-LiBr crystal that was chosen for the EELS experiment. (b) Evaluation of lattice plane distance. The c-distance seems to be increased by electron beam irradiation (theory c=7.04 Å) a) (c), b) PTI-LiBr and PTI-IF EELS spectra. Evaluation of the k-edge yields the N/C ratio are in good agreement with the theoretical expected values of 9N/6C = 1.5. Quantitative analysis of the ion species is not

possible due to the overlap of the Br-M4,5 edge at 69 eV with the Li k-edge at around 62 eV. b) EELS spectrum comparison of PTI-LiBr and PTI-IF attesting absence of Li ions in PTI-IF.

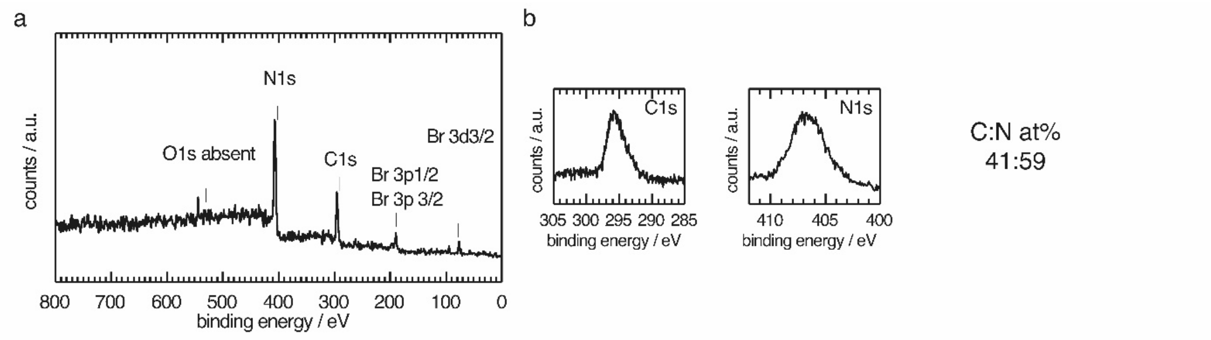

Figure S 3. XPS spectra of PTI-LiBr from new synthesis conditions reveal a further decrease in carbonization when compared to our last work. a) Survey spectra show presence of carbon nitrogen and bromine. b) Quantification of carbon and nitrogen single element spectra reveal a C:N ratio of 41:59 at%.

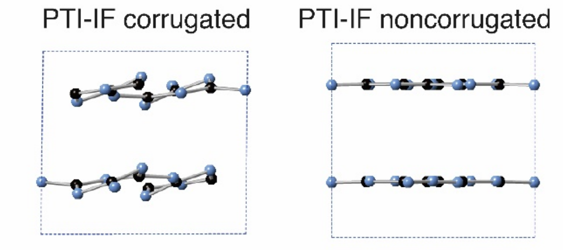

Figure S 4. Comparison of corrugated and non corrugated b-achsis view of PTI AA* stacked cif files used for Pawley refinement.

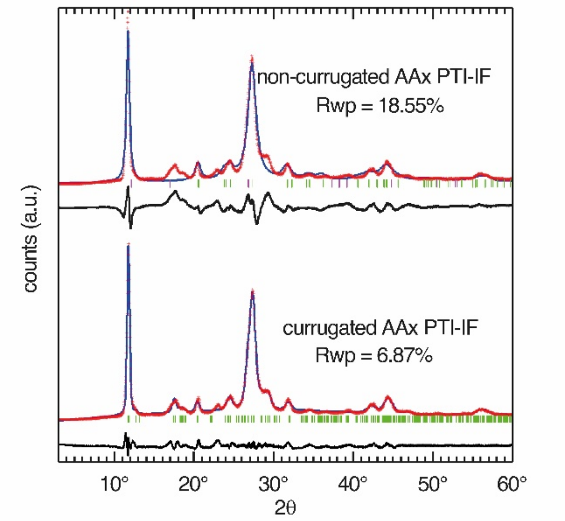

Figure S 5. Comparison of Pawley refinement of PTI-IF AA* stacked unit cells with and without layer corrugation. Red crosses indicate the experimental spectrum, the blue line is the result of the Pawley fit, green markers show markers for observed reflections and violet markers show positions of symmetry forbidden reflections. Pawley refinement of the diffractogram of the "flat" non corrugated unit cell reveals several symmetry forbidden reflections (violet markers) due to the high symmetry of the unit cell (P63CM, Spacegroup 185). The relaxation of the system via DFT induces layer corrugation which in turn reduces the symmetry of the system to P1, hence no symmetry forbidden reflections are observed in the calculation result. Intensities at positions with prior absences are indeed observed for example at 12.2° and 17.1°. Additionally the peaks at 18.5° and 29.0° are better represented. A peak that is not accounted for in any structure we used for Pawley refinement is at 23.0°. The Pawley refinement with the corrugated unit cell overall better fits the observed spectrum as is also reflected by the lower weighted profile R-value (Rwp).

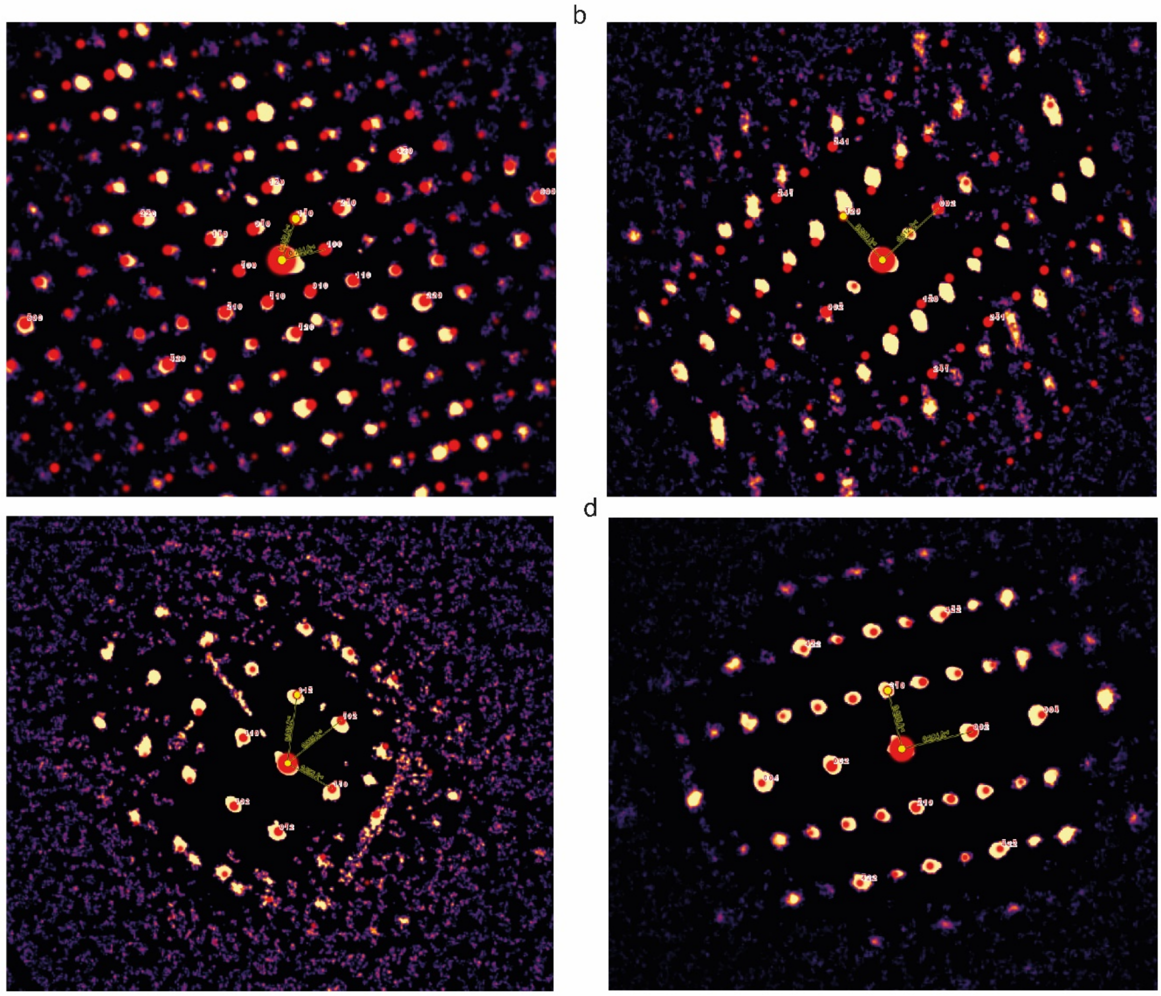

Figure S 6. Overlay of CBED diffractograms of PTI-IF (a 001, b 100) and PTI-LiBr (c 001, d 100) with calculated patterns (red dots). Systematic absences are absent in the 100 pattern of PTI-IF.

Partial charges (Bader)

PTI-IF PTI-LiBr

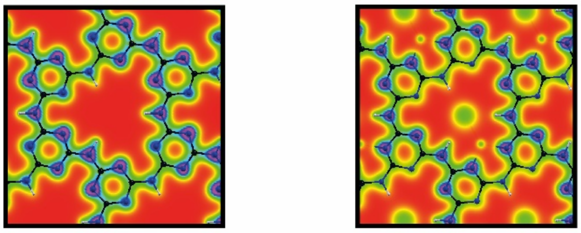

species	charge
Triazine 1 / 2	+2.32e / +2.05e
bridge 1 / 2 / 3	-1.42e / -1.57e / -1.39e

species	charge
Triazine 1 / 2	+1.61e / +1.09e
bridge 1 / 2 / 3	-0.77e / -0.69e / -1.34e
Li	+0.87e
Br	-0.77e

Figure S 7. Partial charge analysis (Bader) of PTI-IF and PTI-Br. Electron density at carbon and nitrogen is higher in PTI-LiBr when compared to PTI-IF. Carbon is represented as black sphere, nitrogen blue sphere and hydrogen white sphere. Lithium and bromine are not represented as spheres. Bromine is positioned in the center of the pore in PTI-LiBr and Lithium is off centered.

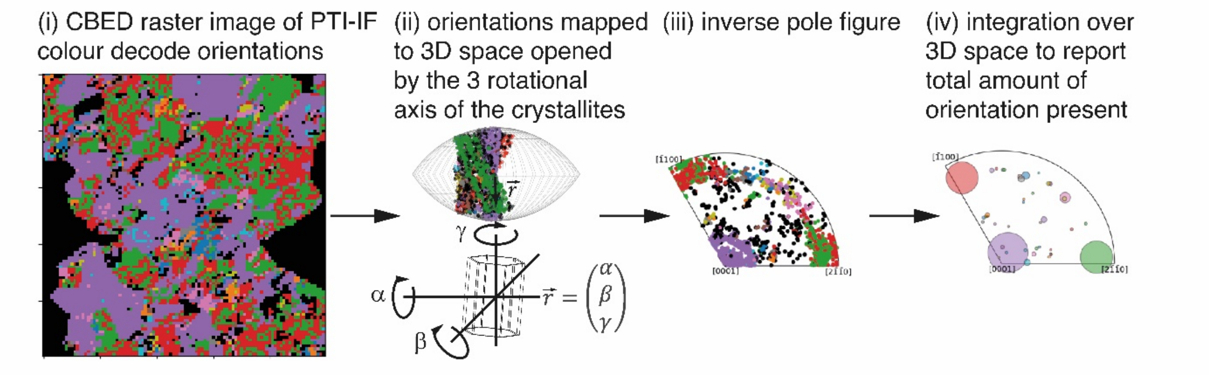

Figure S 8. (i) Each individual pixel represents one CBED pattern that was matched to the orientation of the crystal structure. The orientations are mapped into 3D-space utilizing the three rotational axis of the crystal relative to the electron beam that define three integers giving rise to the length and orientation of a vector in the 3D space with the 0001/001 zone axis being the center (ii). Each vector is then represented by a point in 3D space (iii). Pixels with similar orientation are then summed over the 3D space to give a 2D representation (iv).

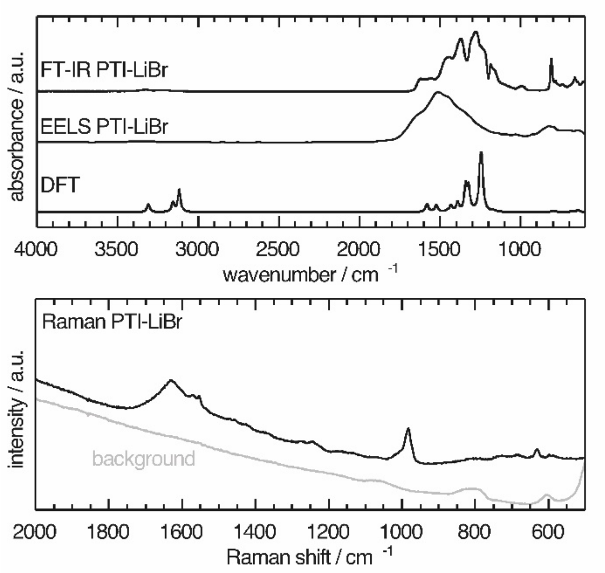

Figure S 9. Vibrational modes in PTI-LiBr determined by FT-IR, DFT, Raman (266 nm) and EELS.

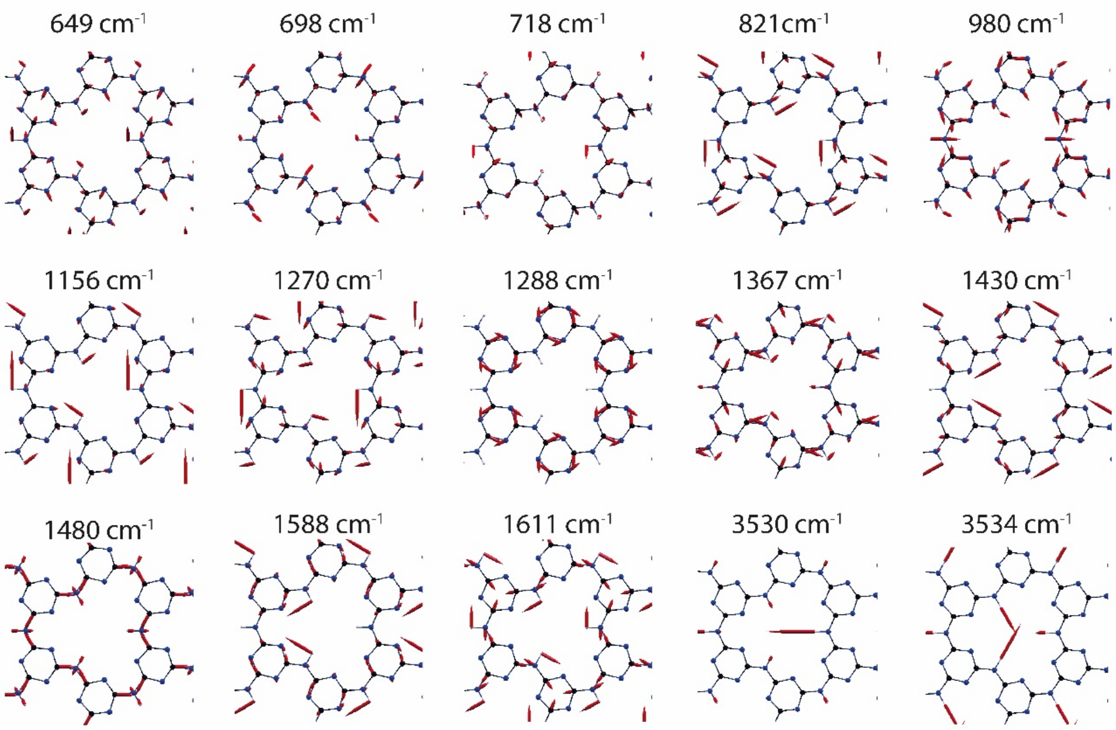

experimentally observed IR	Raman	EELS	ν calc.	rel. int. IR	assignment
			3530, 3534		NH stretching
3360 (b, w)					intercalated water
1620 (m)	1633 (b)	1653 (b)	1611	0.00	NH wagging+ring breathing
1590 (m)	1562 (b)		1588	11.14	quadrant ringstretch
	1490 (w)	1517 (b)	1480	0.07	NH stretching+NC stretching
1455 (m)	1460 (w)	1436 (b)	1430	52.93	NH wagging+ring deformation
1367 (s)	1375 (m)	1333 (b)	1367	95.78	NH wagging+ring deformation
1270 (vs)	1277 (w)		1288	0.11	ring deformation
	1254 (w)		1270	49.74	NH wagging+ring deformation
1182 (w)			1156	1.2	NH wagging+ring deformation
982 (w)	990 (vs)		980	0.21	quadrant ringstretch
802 (s)		820 (b)	821	1.41	NH wagging+ring breathing
	733 (vw)		718	0.03	out of plane def.
	692 (vw)		698	1.44	NH wagging+out of plane ring breathing
642 (s)	650 (vw)	667 (b)	649	0.46	ring deformation

Figure S 10. Phonon normal modes of PTI-IF monolayer. Top: phonon frequencies and corresponding eigenmodes represented as red arrows. The normal modes are mainly composed by N-H and C-N stretching. Bottom: summary of DFT vibrational analysis of a monolayer compared to FT-IR, Raman, and EELS results.

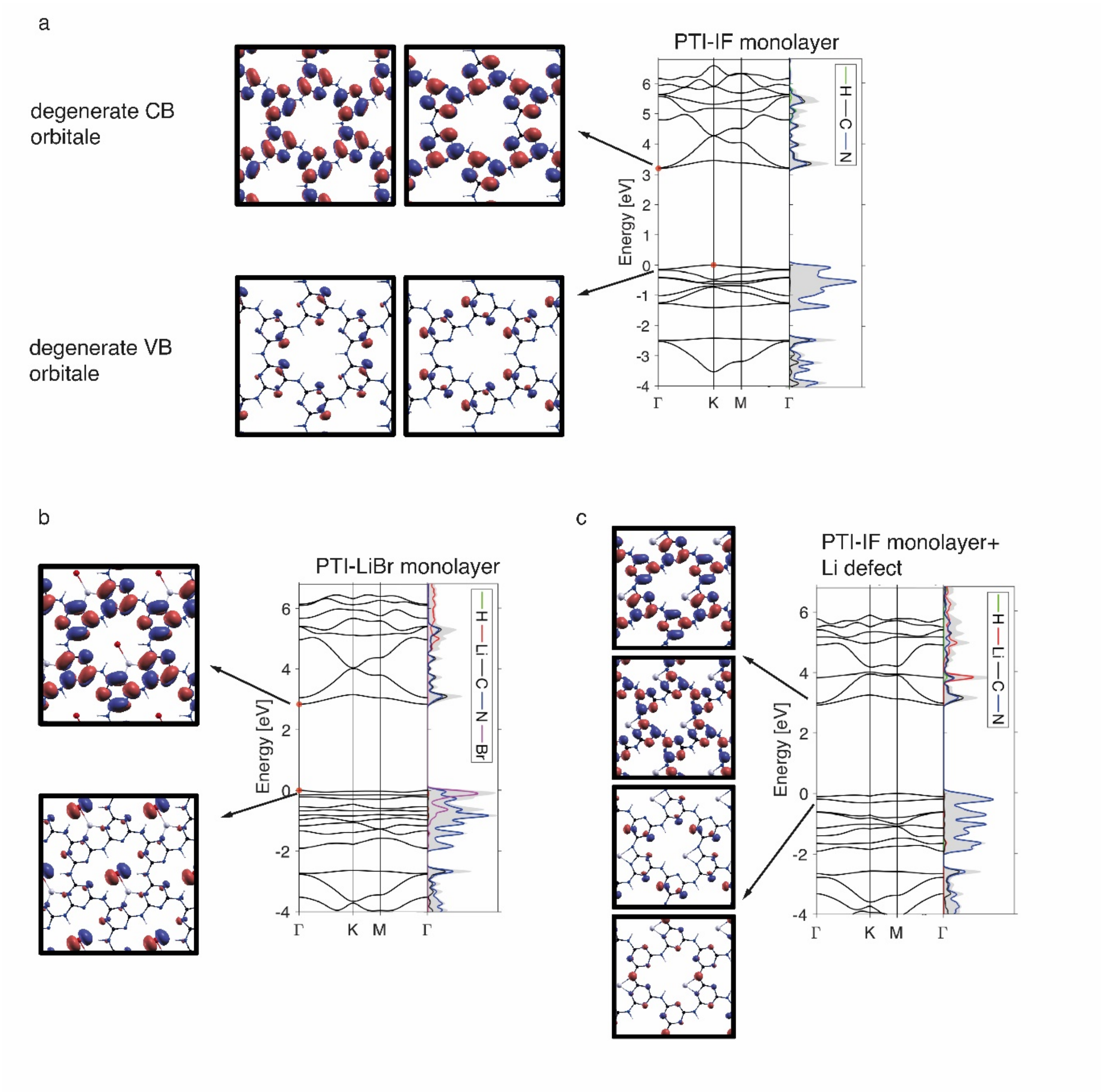

Figure S 11. a) PTI-IF pristine monolayer electronic bandstructure and isosurfaces of the double degenerate valence band and conduction band Bloch states at Γ point (i.e., BZ centre). b) PTI-LiBr monolayer electronic bandstructure and isosurfaces of the valence band and conduction band Bloch states at Γ point. c) Effect of Lithium defect replacing one Hydrogen at the imide bridge in the PTI-IF monolayer.

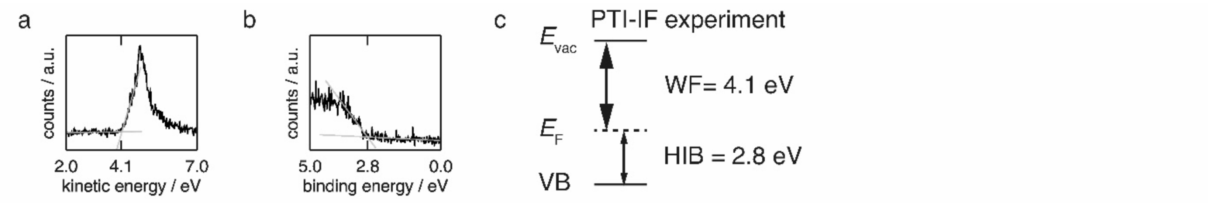

Figure S 12. Ultraviolet photoelectron spectroscopy (UPS) results for PTI-IF layer on indium tin oxide. a) Secondary electron cut-off. b) Valence band scan. c) Energy levels of PTI-IF obtained from UPS. The ionization potential (IP) is equal to IP=WF+HIB=6.9 eV. With work function denoted by WF and hole injection barrier by HIB.

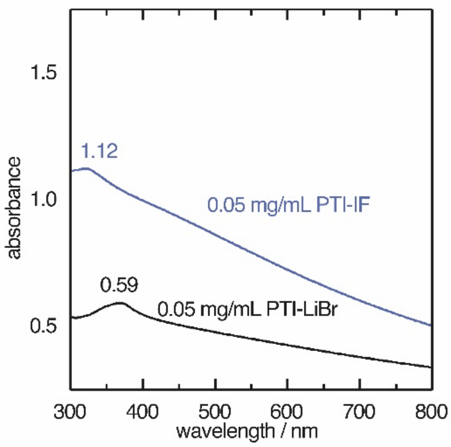

Figure S 13. Absorption of PTI-IF (blue) and PTI-LiBr (black) emulsions. Molar absorption coefficients: 9032 $M^{-1}cm^{-1}$ PTI-IF, 6860 $M^{-1}cm^{-1}$ PTI-LiBr.

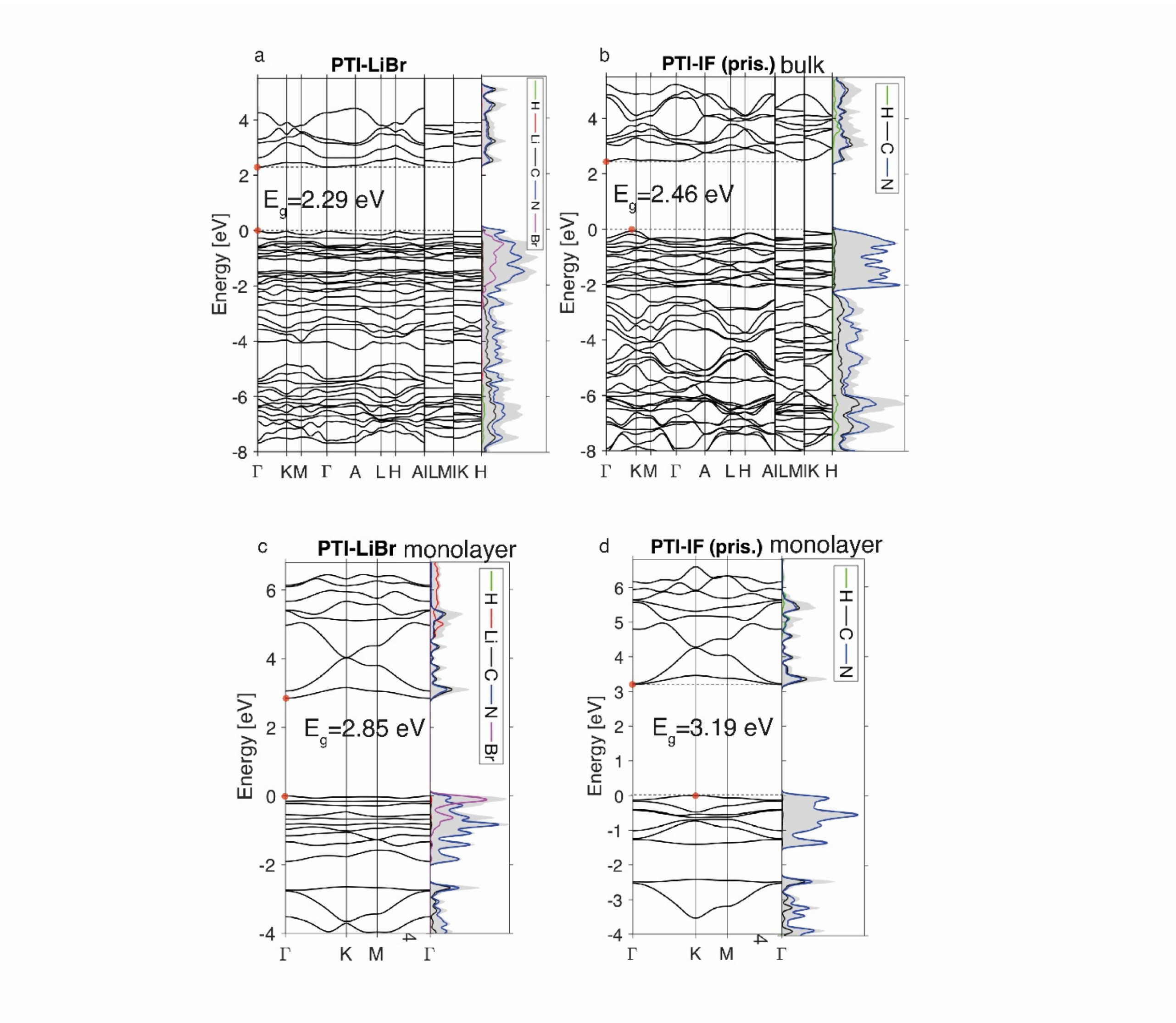

Figure S 14. Calculated band structures of a) PTI-LiBr AA* bulk, b) PTI-IF AA* bulk, c) PTI-LiBr monolayer and d) PTI-IF monolayer.

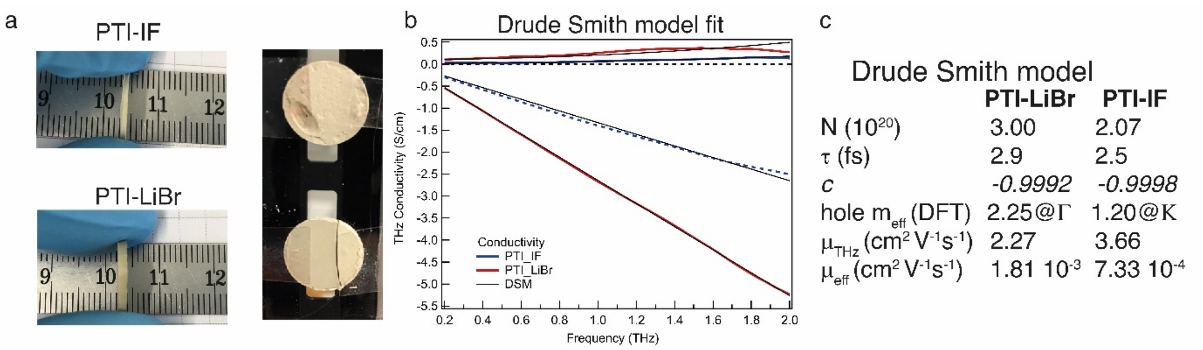

Figure S 15. a) Pressed PTI pellets for THz spectroscopy. b) Drude Smith model fit. c) Drude smith fitting parameters.

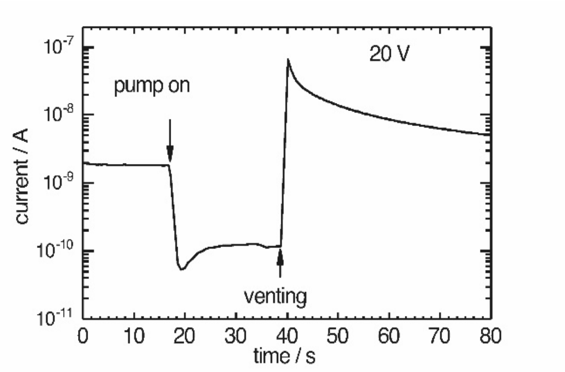

Figure S 16. Current versus time graph of PTI-IF interdigitated device in vacuum chamber. Reduction of pressure leads to drop in current while venting to atmospheric pressure leads to current flow.

References

[1] A. Tkatchenko, R. A. Distasio, R. Car, M. Scheffler, *Phys. Rev. Lett.* **2012**, *108*, 1.

[2] J. P. Perdew, K. Burke, M. Ernzerhof, *Phys. Rev. Lett.* **1996**, *77*, 3865.

[3] A. Tkatchenko, M. Scheffler, *Phys. Rev. Lett.* **2009**, *102*, 6.

[4] P. Giannozzi, S. Baroni, N. Bonini, M. Calandra, R. Car, C. Cavazzoni, D. Ceresoli, G. L. Chiarotti, M. Cococcioni, I. Dabo, A. Dal Corso, S. De Gironcoli, S. Fabris, G. Fratesi, R. Gebauer, U. Gerstmann, C. Gougoussis, A. Kokalj, M. Lazzeri, L. Martin-Samos, N. Marzari, F. Mauri, R. Mazzarello, S. Paolini, A. Pasquarello, L. Paulatto, C. Sbraccia, S. Scandolo, G. Sclauzero, A. P. Seitsonen, A. Smogunov, P. Umari, R. M. Wentzcovitch, *J. Phys. Condens. Matter* **2009**, *21*.

[5] S. J. Clark, M. D. Segall, C. J. Pickard, P. J. Hasnip, M. I. J. Probert, K. Refson, M. C. Payne, *Zeitschrift fur Krist.* **2005**, *220*, 567.

[6] D. Johnstone, P. Crout, Pyxem https://doi.org/10.5281/zenodo.2649351.

[7] F. de la Peña, V. Tonaas Fauske, Hyperspy https://doi.org/10.5281/zenodo.5608741.

[8] D. N. Johnstone, B. H. Martineau, P. Crout, P. A. Midgley, A. S. Eggeman, *J. Appl. Crystallogr.* **2020**, *53*, 1293.

[9] D. E. Soule, *Phys. Rev.* **1958**, *112*, 698.

[10] K. Sugihara, K. Kawamuma, T. Tsuzuku, *J. Phys. Soc. Japan* **1979**, *47*, 1210.

[11] N. Kumar, J. He, D. He, Y. Wang, H. Zhao, *J. Appl. Phys.* **2013**, *113*, 1.

[12] E. Jin, K. Geng, S. Fu, S. Yang, N. Kanlayakan, M. A. Addicoat, N. Kungwan, J. Geurs, H. Xu, M. Bonn, H. I. Wang, J. Smet, T. Kowalczyk, D. Jiang, *Chem* **2021**, *7*, 3309.

[13] M. Wang, M. Ballabio, M. Wang, H. Lin, B. P. Biswal, X. Han, S. Paasch, E. Brunner, P. Liu, M. Chen, M. Bonn, T. Heine, S. Zhou, E. Cánovas, R. Dong, X. Feng, *J. Am. Chem. Soc.* **2019**, *141*, 16810.

[14] S. Fu, E. Jin, H. Hanayama, W. Zheng, H. Zhang, L. Di Virgilio, M. A. Addicoat, M. Mezger, A. Narita, M. Bonn, K. Müllen, H. I. Wang, *J. Am. Chem. Soc.* **2022**, *144*, 7489.

[15] R. Dong, P. Han, H. Arora, M. Ballabio, M. Karakus, Z. Zhang, C. Shekhar, P. Adler, P. S.

Petkov, A. Erbe, S. C. B. Mannsfeld, C. Felser, T. Heine, M. Bonn, X. Feng, E. Cánovas, *Nat. Mater.* **2018**, *17*, 1027.
[16] V. I. Arkhipov, P. Heremans, E. V. Emelianova, G. J. Adriaenssens, H. Bässler, *Appl. Phys. Lett.* **2003**, *82*, 3245.
